# Supplementary material for: Occurrence of shigellosis in pediatric diarrheal patients in Chattogram, Bangladesh: A molecular based approach
Source: PLoS One. 2023 Jun 15;18(6):e0275353. doi: 10.1371/journal.pone.0275353 (PMC10270574; doi:10.1371/journal.pone.0275353)
Supplement: S1 Table — (PDF) [file pone.0275353.s002.pdf]

Supporting Information

Occurrence of Shigellosis in Pediatric Diarrheal Patients in Chattogram, Bangladesh: A Molecular Based Approach

A K M Zakir Hossain[1¶], Md. Zahid Hasan[1], Sohana Akter Mina[1], Nahid Sultana[2], A M Masudul Azad Chowdhury[*1¶]

1. Laboratory of Microbial & Cancer Genomics, Department of Genetic Engineering & Biotechnology, University of Chittagong, Bangladesh

2. Department of Microbiology, Chattogram Maa-O-Shishu Hospital Medical College, Chattogram, Bangladesh

* Corresponding author

E-mail: masud.geb@cu.ac.bd

¶ These authors contributed equally to this work.

Table S1. Occurrence *of E.coli/ Klebsiella, Pseudomonas aeruginosa, Salmonella paratyphi, Salmonella typhi, Shigella spp, and Shigella flexneri* among diarrheic male and female children under-5 years of age in Chattogram, Bangladesh.

Variables		Frequency	Percent (%)	Sex		Significance
				Male	Female	
Microorganisms	*E.coli/ Klebsiella*	97	47.5	37	60	p<0.01
	Pseudomonas aeroginosa	5	2.5	1	4	
	Salmonella paratyphi	71	34.8	27	44	
	Salmonella typhi	2	1.0	0	2	
	Shigella spp	2	1	0	2	
	Shigella flexneri	27	13.2	12	15	
Shigellosis	Negative	175	85.8	110	65	p<0.01
	Positive	29	14.2	12	17	
Age	1D ≤ 6M	32	15.7	20	12	p<0.01
	7M ≤ 1Y	94	46.1	59	35	
	1Y 1M ≤ 5Y	72	35.3	45	27	
	5Y <	6	2.9	3	3	
