# Supplementary material for: Occurrence of shigellosis in pediatric diarrheal patients in Chattogram, Bangladesh: A molecular based approach
Source: PLoS One. 2023 Jun 15;18(6):e0275353. doi: 10.1371/journal.pone.0275353 (PMC10270574; doi:10.1371/journal.pone.0275353)
Supplement: Attachment — Submitted filename: Reviwer Comments.pdf [file pone.0275353.s003.pdf]

Prevalence of Shigellosis in Pediatric Diarrheal Patients in Chattogram, Bangladesh: A Molecular Based Approach is an interesting study with respect to improving the accurate identification and the treatment of the most severe and alarming shigellosis. The manuscript is ok and accepted for publication after incorporation of some minor comments.

1) Rephrased the title as "Prevalence" is a broad term. Authors only collected samples from Chittagong Medical College Hospital while no data/information about Shigellosis from other hospitals in Chittagong was provided in the study.

2) Authors need to improve introduction section providing more background knowledge about the problem statement using relevant references.

3) Molecular identification is a well-known technique for the identification of organisms, but it is a time-consuming process as compared to other fast diagnostic and identification techniques for Shigellosis in diarrheal patients. Better to justify this in the introduction section and also add some background literature about other traditional diagnostic techniques for shigellosis in

4) Correct the word "Gram-negative" instead of gram-negative throughout manuscript.

5) Line 63: it was concluded instead of We concluded

6) Line 65: remove the word we show and also rephrase the sentence.

7) Line 68: rephrase the first line and start the paragraph like that " In the current student, human clinical samples were investigated therefore, ethical approval was taken from..............................and so on.

8) For molecular identification, PCI method is mostly used for extraction of DNA, but in the current study, authors used boiling method, so can author justify it why not using PCI method for extraction of DNA?

9) Discussion section of the manuscripts needs to be improved by comparing results with already published literature. More relevant references are needed to be incorporated in the discussion section to justify the results.

10) Some of the references are too old. better to update references (not more than 05 years old).
