# Supplementary material for: Occurrence of shigellosis in pediatric diarrheal patients in Chattogram, Bangladesh: A molecular based approach
Source: PLoS One. 2023 Jun 15;18(6):e0275353. doi: 10.1371/journal.pone.0275353 (PMC10270574; doi:10.1371/journal.pone.0275353)
Supplement: Attachment — Submitted filename: Response to Reviewers.pdf [file pone.0275353.s005.pdf]

Response to Reviewers

Reviewer-1 Comments' Reply

Thank you for taking the time to review our manuscript titled "**Prevalence of Shigellosis in Pediatric Diarrheal Patients in Chattogram, Bangladesh: A Molecular Based Approach**." We appreciate your valuable feedback, which helped us to improve the manuscript. We are glad to know that you found our study interesting and important in improving the accurate identification and treatment of shigellosis. We are grateful for your time and effort in reviewing our manuscript and providing constructive feedback, which has undoubtedly improved the quality of our work. We have revised the manuscript and tried to make corrections according your suggestion. The addition and correction were highlighted with yellow mark.

1) Rephrased the title as "Prevalence" is a broad term. Authors only collected samples from Chittagong Medical College Hospital while no data/information about Shigellosis from other hospitals in Chittagong was provided in the study.

Answer: Thank you for your suggestion to rephrase the title. We agree that "prevalence" is a broad term and have changed it to "Occurrence" to better reflect the scope of our study. Additionally, we understand your concern regarding the sample collection from only one hospital in Chittagong. We would like to clarify that the Chittagong Ma-O-Shishu Hospital Medical College is a major referral hospital for pediatric patients in the Chittagong region, which is why we have chosen to collect our samples from this hospital. While we acknowledge that data from other hospitals in the region would have provided a more comprehensive picture. However, we believe that our study still provides valuable insights into the occurrence of Shigellosis in this particular hospital setting,

and future studies could expand upon our findings by including data from other hospitals in the region.

2. Authors need to improve introduction section providing more background knowledge about the problem statement using relevant references.

Answer: We appreciate your suggestion to improve the introduction section of our manuscript. We have carefully revised and updated the introduction section by incorporating more relevant references and providing additional background knowledge about the problem statement. We hope the revised version now adequately justifies the significance and relevance of our study.

3) Molecular identification is a well-known technique for the identification of organisms, but it is a time-consuming process as compared to other fast diagnostic and identification techniques for Shigellosis in diarrheal patients. Better to justify this in the introduction section and also add some background literature about other traditional diagnostic techniques for shigellosis

Answer: Thank you for your valuable comment. We have added additional information in the introduction section to provide a better understanding of traditional diagnostic techniques for shigellosis. We have also highlighted the advantages of molecular identification techniques in comparison to traditional diagnostic techniques for Shigellosis. We believe that these additions will improve the overall quality of the manuscript and address your concerns

4) Correct the word "Gram-negative" instead of gram-negative throughout manuscript.

Answer: gram-negative was replaced by **"Gram-negative"**

5) Line 63: it was concluded instead of We concluded

Answer: Correction was done.

6) Line 65: remove the word we show and also rephrase the sentence.

Answer: Done.

7) Line 68: rephrase the first line and start the paragraph like that " In the current student, human clinical samples were investigated therefore, ethical approval was taken from............................and so on.

Answer: Done

8) For molecular identification, PCI method is mostly used for extraction of DNA, but in the current study, authors used boiling method, so can author justify it why not using PCI method for extraction of DNA?

Answer: The boiling method for DNA extraction is a simple, quick, and cost-effective technique that has been shown to yield high-quality DNA for molecular identification in previous studies (Mina et al. 2023)(Ahmed* and Dablool 2017). In our study, we used the boiling method due to financial constraints as the PCI method requires specialized equipment and reagents that are relatively expensive. Moreover, the boiling method is more convenient for field-based studies and can be easily performed in remote settings where resources are limited. Despite being a cost-effective method, we ensured the quality and quantity of the extracted DNA by using a NanoDrop to measure the DNA concentration and purity, which was found to be satisfactory for downstream PCR amplification. Therefore, we are confident that our decision to use the boiling method for DNA extraction was appropriate, given its practicality and cost-effectiveness within the context of our study

9) Discussion section of the manuscripts needs to be improved by comparing results with already published literature. More relevant references are needed to be incorporated in the discussion section to justify the results

Thank you for your valuable comments and suggestions. We have carefully considered your feedback and have made significant improvements to the discussion section of our manuscript. We have incorporated more relevant references to compare our results with those of previously published literature. We believe that these changes have enhanced the significance and validity of our findings, and we appreciate your input in helping us to improve our manuscript.

10) Some of the references are too old. better to update references (not more than 05 years old).

Answer: Thank you for your helpful comments on our manuscript. We have taken your suggestion to update our references seriously, and we have replaced several older references with more recent. We believe that these updated references more accurately reflect the current state of research in the field, and will help to strengthen the impact of our findings.

Reviewer-2 Comments' Reply

The manuscript entitled "Prevalence of Shigellosis in Pediatric Diarrheal Patients in Chattogram, Bangladesh: A Molecular Based Approach has addressed an important and interesting public health issue. I appreciate the authors for conducting research within limited resources. However, the manuscript is not publishable in current form and is here by rejected due to following points.

- **Weak theoretical framework and literature review.**
- **The abstract is poorly written and there is no reflection of significant results.**
- **The introduction, methodology, results and discussion are not organized.**
- **The data is weak and non significant**

- **there is a complete mismatch between the theoretical framework and the empirical analysis.**
- **A lot of grammatical mistakes are there in article**
- **No novelty**

Answer: Thank you for your review and constructive feedback. We appreciate your acknowledgement of our research efforts despite the limitations of our resources. Regarding the points raised in your review, we have carefully considered your feedback and have made significant revisions to address the weaknesses you have highlighted.

We have improved the theoretical framework and literature review by providing more relevant and up-to-date references. We have also rewritten the abstract to reflect the significant findings of our study. Additionally, we have reorganized the introduction and discussion sections to improve the overall flow and coherence of the manuscript.

To address concerns about the data, we have re-analyzed our results and provided more in-depth explanations of our findings. Furthermore, we have strengthened the link between the theoretical framework and the empirical analysis, and have thoroughly proofread the manuscript to eliminate grammatical errors.

While we understand your concern about novelty, we believe that our study's molecular-based approach to identifying the prevalence of *Shigella spp.* in pediatric diarrheal patients in Chattogram, Bangladesh, is a significant contribution to the field. Our study's findings shed light on the prevalence and potential transmission of multi-drug-resistant strains of *S. flexneri* in a developing country, which has important implications for public health interventions.

We hope that our revisions address your concerns and that you will consider our manuscript for publication in the esteemed journal.
