# Supplementary material for: Occurrence of shigellosis in pediatric diarrheal patients in Chattogram, Bangladesh: A molecular based approach
Source: PLoS One. 2023 Jun 15;18(6):e0275353. doi: 10.1371/journal.pone.0275353 (PMC10270574; doi:10.1371/journal.pone.0275353)
Supplement: Attachment — Submitted filename: Response to Reviewers.pdf [file pone.0275353.s006.pdf]

# Response to Reviewers

## Response to Reviewer 1:

We would like to express our sincere gratitude for your insightful review of our article. Your feedback was incredibly valuable in helping us to improve the quality and clarity of our work.

Your positive comments and constructive criticism were particularly appreciated, and we are grateful for the time and effort you put into reviewing our manuscript. Your expertise in the field was evident in your thorough and thoughtful review, and we are fortunate to have had the opportunity to benefit from your insights.

Once again, thank you for your time and effort in reviewing our article. Your feedback has been incredibly helpful, and we are grateful for your contribution to our work.

## Response to Reviewer 2:

Thank you for your review and constructive feedback. We appreciate your acknowledgement of our research efforts despite the limitations of our resources. Regarding the points raised in your review, we have carefully considered your feedback and have made significant revisions to address the weaknesses you have highlighted.

**Improve the results particularly headings.**

**Once positive control has been mentioned in methodology then there is no need to repeat and mention control and accession number again and again in results and discussion.**

**Answer:** We have carefully considered the feedback and made the necessary revisions to our manuscript. Specifically, we have improved the results section by ensuring that the headings are more informative and accurately reflect the content of each subsection.

Moreover, we have taken into account the reviewer's suggestion to avoid redundant language. We have removed unnecessary repetition of the positive control, control, and accession numbers in the results and discussion sections.

**Line 137: Correct the heading. the authors have not worked on prevalence of diarrhoea infections. The phrase seems confusing and misleading**

**Answer:** Done

**Line 146: age range not rang.. correct spelling.**

**Answer:** Done

**Revise the table No 2**

**Revise the title. If this is main table of your results, i suggest, there is no need to mention all the bacteria in title as your manuscript has focused on shigellosis only. Please delete miscellaneous bacteria from the table. mention the abbreviations in the caption/legend of the table.**

**Answer:** Done

**Improve Figures 1-4. Figures should be self-explanatory. So revise them taking help of bio-statistian.**

**Answer:** We have revised Figures 1-4 to make them more self-explanatory. We have added more labels and captions to help readers better understand the figures without having to refer back to the text. We hope that the new figures will be clearer and more informative for readers.

**Revise and relabel the figure 2 showing gel. The numbering 1, 2,3.... should be from left to right rather than ........3,2,1. Just mention the clinical sample instead of repeating *S. flexneri* isolate number in each lane.**

**Answer:** We have revised the figure 2 as per your recommendation, with the numbering 1, 2, 3... from left to right. We have also relabeled the figure to show only the clinical sample without repeating the *S. flexneri* isolate number in each lane, making it more clear and self-explanatory. We appreciate your feedback and believe that the revised figure has improved the quality of our manuscript.

**In figure 4 correct the title, What is meant by E.coli/klebsiella as both are two different bacteria. Write them separately.**

**Answer:** Done.

**Improve the description in discussion.**

**Answer:** We have carefully revised the discussion section, as suggested. We have improved the description to provide a more comprehensive and accurate interpretation of our results.

**Line No 194: mention the year with citation.**

**Answer:** Done.

**Genus should be written in Italics.**

Answer: Done.

**Add proper conclusion, recommendations and limitations of this study at the end of discussion section.**

**Answer:** Thank you for your valuable feedback and suggestions. We have revised the discussion section by adding a proper conclusion that summarizes the main findings of the study. We have also included recommendations for future research in this field and limitations of our study to provide a more comprehensive understanding of our work.

We appreciate your time and effort in reviewing our manuscript, and we believe that your suggestions have significantly improved the quality of our work.
