# Supplementary material for: Occurrence of shigellosis in pediatric diarrheal patients in Chattogram, Bangladesh: A molecular based approach
Source: PLoS One. 2023 Jun 15;18(6):e0275353. doi: 10.1371/journal.pone.0275353 (PMC10270574; doi:10.1371/journal.pone.0275353)
Supplement: Attachment — Submitted filename: Shigellosis in Bangladesh review.docx [file pone.0275353.s004.docx]

The manuscript entitled “Prevalence of Shigellosis in Pediatric Diarrheal Patients in Chattogram, Bangladesh: A Molecular Based Approach has addressed an important and interesting public health issue. I appreciate the authors for conducting research within limited resources. However, the manuscript is not publishable in current form and is here by rejected due to following points.

- Weak theoretical framework and literature review.
- The abstract is poorly written and there is no reflection of significant results.
- The introduction, methodology, results and discussion are not organized.
- The data is weak and non significant
- there is a complete mismatch between the theoretical framework and the empirical analysis.
- A lot of grammatical mistakes are there in article
- No novelty
